# A Review of Pityriasis Rubra Pilaris and Rheumatologic Associations

**DOI:** 10.1080/10446670410001670008

**Published:** 2004-03

**Authors:** Happy Chan, Fu Tong Liu, Stanley Naguwa

**Affiliations:** 1 Department of Internal Medicine University of California Davis USA; 2 Department of Dermatology University of California Davis USA; 3 Department of Rheumatology University of California Davis USA

## Abstract

Pityriasis rubra pilaris (PRP) is a rare group of hyperkeratotic, papulosquamous disease that can be acquired or inherited. There have been reported cases of rheumatologic associations, mainly arthritis and dermatomyositis. In this review article, we will explore the clinical presentation and classification, rheumatologic associations and treatment modalities of PRP. In addition, we will also report a case of PRP with seronegative arthritis.


*Clinical & Developmental Immunology*, March 2004, Vol. 11 (1), pp. 57–60

Taylor & Francis healthsciences

### A Review of Pityriasis Rubra Pilaris and Rheumatologic Associations

HAPPY CHAN[a], FU TONG LIU[b] and STANLEY NAGUWA[c,*]

[a]*Department of Internal Medicine, University of California, Davis, USA;* [b]*Department of Dermatology, University of California, Davis, USA;* [c]*Department of Rheumatology, University of California, Davis, USA*

Pityriasis rubra pilaris (PRP) is a rare group of hyperkeratotic, papulosquamous disease that can be acquired or inherited. There have been reported cases of rheumatologic associations, mainly arthritis and dermatomyositis. In this review article, we will explore the clinical presentation and classification, rheumatologic associations and treatment modalities of PRP. In addition, we will also report a case of PRP with seronegative arthritis.

*Keywords*: Pityriasis rubra pilaris; Review; Arthritis; Rheumatologic; Treatment

### CASE REPORT

A 50-year-old East Indian male with a 2-year history of nonspecific knee arthritis presented to his primary care doctor with a new scaly and pruritic rash. It began on the extensor surfaces of the elbow and bilaterally spread onto the forearms. Similar lesions began to appear over the scalp, trunk, axilla and knee. His nails became thickened and hyperpigmented. The patient was diagnosed with psoriasis. With the appearance of the lesions, his knee pain intensified, especially when ascending the stairs. The pain would awaken him at night. During this period, he had stiffening of his extremities. Besides fatigue, he denied any recent acute illnesses, constitutional symptoms, and mucocutaneous or ocular lesions. His past medical history included hypothyroidism and seasonal allergies. He denied food, drug and environmental allergies. His medication list at presentation included levothyroxine and glucosamine and chondroitin. He smoked tobacco occasionally and owned a cat. He had not traveled outside the country recently. His mother had nonspecific arthritis. On physical examination, his vitals were normal. There were no lymphadenopathy, oral lesions and hepatosplenomegaly. Neither joint deformity nor synovitis was appreciated. He had tenderness of the right knee on palpation with mild patellar laxity. He had multiple sharply demarcated red–orange scaly plaques over the extensor surfaces of the forearms, axillae, knees and trunk. The plaques were nearly confluent on his trunk with the exception of islands of normal skin. The skin of his palms was thick and dry. Bilaterally, his second and third fingernails were hyperkeratotic with pitting and hyperpigmentation. X-rays of the hands and knees were normal. Complete blood count, complete metabolic panel and urinalysis were normal. The erythrocyte sedimentary rate was 15; antinuclear antibody was 1:160 with a nucleolar pattern. Thyroid stimulating hormone, immunoglobulin and complement levels were normal. Rheumatoid factor, hepatitis panel and RPR were negative. He did not respond to the conventional psoriatic treatment with ultraviolet light therapy UVA and UVB. Eventually, a punch biopsy was performed, and a diagnosis of pityriasis rubra pilaris (PRP) was made. The patient was placed on methotrexate therapy and his arthritis and skin lesions subsequently resolved.

### INTRODUCTION

PRP is a rare group of hyperkeratotic, papulosquamous disease that can be acquired or inherited. It was first described by Alaudius Tarral in 1828, and Devergie named it "pityriasis pilaris" in 1856 (Griffiths, 1976; Fox *et al.*, 1985). In 1889, Besnier renamed the disease to PRP (Griffiths, 1976). This rare skin disorder occurs in all races, affects both sexes equally, and has been reported world wide (Griffiths, 1980). The incidence of patients presenting to dermatology clinics in the United States has been reported to be 1 in 3500–5000 patients (Rinker and Shenefelt, 2003). PRP occurs in a bimodal distribution, peaking in the first and then the fifth decades (Griffiths, 1980). Although there are hereditary forms, most cases are acquired. It is commonly confused with other papulosquamous and erythrodermic disorders, particularly psoriasis. Although the etiology is unknown, there has been recent

*Corresponding author.

ISSN 1740-2522 print/ISSN 1740-2530 online © 2004 Taylor & Francis Ltd
DOI: 10.1080/10446670410001670008

TABLE I Differential diagnosis of pityriasis rubra pilaris (Griffiths, 1980; Fox *et al.*, 1985; Caplan *et al.*, 1997; Albert and Mackool, 1999)

- Cutaneous T cell lymphoma
- Dermatomyositis
- Dermatophytosis
- Eczematous dermatitis
- Erythrokeratoderma variabilis
- Erythrokeratoderma Variabilis
- Follicular eczema
- Follicular ichthyosis
- Generalized hypersensitivity reaction
- Lichen Planopilarus
- Mycosis Fungoides
- Parasoriasis
- Psoriasis
- Seborrheic dermatitis
- Secondary syphilis
- Subacute cutaneous lupus erythematosus
- Vitamin A deficiency discussion that it may be due to immune system dysregulation where there is an abnormal response to antigenic triggers (Albert and Mackool, 1999; Betloch *et al.*, 2001; Behr *et al.*, 2002). However, hypothesis has ranged from vitamin A deficiency to absence of retinol-binding protein. Interestingly, PRP has been associated with malignancy, autoimmune diseases, trauma and infections, particularly human immunodeficiency virus (HIV). Besides the rheumatologic association, several of the autoimmune processes associated with PRP include myasthenia gravis, hypothyroidism and celiac sprue (Albert and Mackool, 1999). Histological features of PRP are nonspecific and diagnosis cannot be made solely on histology (Fox *et al.*, 1985; Cohen and Prystowsky, 1989). However, it helps to exclude disorders with similar characteristics. The differential diagnosis is frequently papulosquamous and erythroidermic disorders (Table I). The nonspecific features include hyperkeratosis, alternating orthokeratosis and parakeratosis in vertical and horizontal directions, irregular acanthosis, a confluent granular layer, and perivascular lymphocytic dermal infiltrate (Cohen and Prystowsky, 1989; Albert and Mackool, 1999; Behr *et al.*, 2002). An important distinguishing feature is acantholysis with focal acantholytic dyskeratosis (Albert and Mackool, 1999). It has been reported that patients with PRP are negative for IgG, IgA, IgM and $C_3$ direct immunofluorescence tests (Albert and Mackool, 1999). Radioimmunoassay of PRR scales contained small amounts of leukotriene $B_4$, $C_{3a}$, $C_{4a}$ and $C_{5a}$, which is in the normal range of noninflammatory skin (Albert and Mackool, 1999). There has been a report of one patient with hypogammaglobulinemia and another patient with activated T suppressor cells and impaired T helper cells (Bonomo *et al.*, 1997; Albert and Mackool, 1999). In this review article, we will explore the clinical presentation and classification, rheumatologic associations and treatment modalities of PRP.

### CLINICAL PRESENTATION AND CLASSIFICATION

The widely accepted classification of PRP was proposed by Griffiths (1980) (Table II). Griffith established five types of PRP based on clinical features, age of onset, and prognosis. Recently, a sixth class was proposed to acknowledge the HIV-associated type of PRP (Miralles *et al.*, 1995). PRP in adults typically spreads in a cephalad to caudal direction, whereas in children it is opposite (Albert and Mackool, 1999). Juvenile types have a tendency to relapse. Systemic symptoms, such as malaise, fatigue, fever and chills can accompany PRP (Rinker and Shenefelt, 2003).

The classic description of PRP found in the literature is of type I PRP. Type I, classical adult PRP, is the most common form where adults are afflicted. As Griffith describes, the elementary lesion is follicular hyperkeratosis (Griffiths, 1976). Progressive erythroderma develops due to follicular hyperkeratosis. Perifollicular erythema becomes confluent with the development of scales. Often there are patches of normal skin interdispersed among the lesions, known as "islands of sparing". The palm and soles become hyperkeratotic and develop an orange hue, which is often very painful and disabling when fissures develop. It is common to find that the dorsal phalanges and extensor aspects of the wrist and thighs will have follicular hyperkertatoic plugs with a consistency of a nutmeg grater. Nail changes are also frequent, consisting of yellow–brown discolorations with splinter hemorrhages and nail plate thickening. With severe disease where there is prolonged facial erythroderma, ectropions can develop.

Type II, atypical adult onset PRP, affects about five percent of the patients (Griffiths, 1980). It is atypical because it can last for more than 20 years. In addition to chronicity, its clinical feature is different from type I by the presence of ichtyosiform scale located on the lower extremities. Alopecia is common. Eczematous changes of the skin are also characteristic. The hyperkeratosis of the palms and soles are more coarse and lamellated.

TABLE II Modified Griffith PRP classification (Griffiths, 1980; Miralles and Nunez, 1996; Misery *et al.*, 1996, Albert and Mackool, 1999)

| Type | Name | Incidence (%) | Distribution | Prognosis (% resolution in 3 years) | Comments |
|---|---|---|---|---|---|
| I | Classic adult | 55 | Generalized | 81 | Most clear in 3 years |
| II | Atypical adult | 5 | Generalized | 20 | Most are chronic |
| III | Classical juvenile | 10 | Generalized | 16 | Most clear in 1 year |
| IV | Circumscribed juvenile | 25 | Localized | 32 | |
| V | Atypical juvenile | 5 | Generalized | 0 | Chronic |
| VI | HIV-associated | Needs more evaluation | Generalized | Needs more evaluation | |

TABLE III Case reports of PRP associated arthritis (Aguilar *et al.*, 1973; Duke *et al.*, 1987; Fiallo *et al.*, 1996; Lister *et al.*, 1997; Conaghan *et al.*, 1999; Nakafusa *et al.*, 2002; Behr *et al.*, 2002)

| Case reports | Year reported | Age | Sex | Rheumatoid factor | Antinuclear antibody | HLA-B27 | Arthritic sites |
|---|---|---|---|---|---|---|---|
| Aguilar *et al.* | 1973 | 62 | M | Unknown | Unknown (LE test was negative) | Unknown | Feet, eventual fingers |
| Duke *et al.* | 1987 | 18 | M | Unknown | Unknown | Unknown | Fingers |
| Fiallo *et al.* | 1996 | 19 | M | Negative | Negative | Positive | Wrist, knees, ankles |
| Lister *et al.* | 1997 | 21 | F | Negative | Positive | Unknown | Neck, fingers, knees, ankles |
| Conaghan *et al.* | 1999 | 25 | M | Negative | Negative | Negative | Shoulders, knees |
| Behr *et al.* | 2002 | 12 | M | Negative | Negative | Unknown | Wrists, fingers |
| Nakafusa *et al.* | 2002 | 53 | M | Positive | Negative | Negative | Neck, shoulders, elbows |
| Chan *et al.* | 2003 | 50 | M | Negative | Positive | Unknown | Knees |

Type III, classical juvenile PRP, is type I PRP occurring in children, usually during the first years of life. Type IV, circumscribed juvenile PRP, is a localized form of PRP, occurring mainly on the elbows, knees and over bony prominences. Type V, atypical juvenile PRP, is a chronic disease and includes most familial forms of PRP.

There has been recognition of type VI, which occurs in established HIV disease presenting with new onset PRP. It is characterized by the development of nodulocycstic and lichen spinulosus lesions (Miralles *et al.*, 1995; Misery *et al.*, 1996). Typically, HIV-associated PRP is refractory to treatment.

### RHEUMATOLOGIC ASSOCIATIONS

Rheumatologic association of PRP is not well defined. Large trials to establish a clear relationship is extremely difficult because PRP is such a rare disease. However, there has been citing of case reports in the literature of PRP associated with arthritis and myositis, specifically dermatomyositis.

PRP association with arthritis was first reported in 1936 (Lister *et al.*, 1997). Between 1973 and 2002, there have been seven case reports describing a temporal relationship of onset of arthritis and PRP (Table III) (Aguilar *et al.*, 1973; Duke *et al.*, 1987; Fiallo *et al.*, 1996; Lister *et al.*, 1997; Conaghan *et al.*, 1999; Behr *et al.*, 2002; Nakafusa *et al.*, 2002). With our report, this will make it the eighth case. Acro-osteolysis has been frequently reported. Four cases have reported that the arthritis preceded the rash whereas three reported the rash to precede the arthritis, and one case reported both to have started at the same time. Treatment for PRP and arthritis varied between authors (Table IV). Treatment of PRP does not guarantee successful treatment of arthritis and vice versa.

Interestingly, five out of the eight cases have been associated with seronegative arthritis and only one was rheumatoid factor positive. The status of the other two cases is unknown. Seronegative arthritis is a heterogeneous class of inflammatory arthritis that is defined by a negative rheumatoid factor test. The diseases classified as seronegative arthritides include psoriatic arthritis, ankylosing spondylitis, Bechet's syndrome, and Reiter's disease. In the cases of PRP associated seronegative arthritis, it has been

TABLE IV Treatment modalities of PRP and arthritis (Aguilar *et al.*, 1973; Duke *et al.*, 1987; Fiallo *et al.*, 1996; Lister *et al.*, 1997; Conaghan *et al.*, 1999; Behr *et al.*, 2002; Nakafusa *et al.*, 2002)

| Case reports | PRP treatment modalities | Arthritis treatment modalities | Failed treatments |
|---|---|---|---|
| Aguilar *et al.* | Vitamin A, Prednisone | Chloroquine, Asprin, Prednisolone | |
| Duke *et al.* | Unknown | Unknown | |
| Fiallo *et al.* | Etretinate | Etretinate | |
| Lister *et al.* | Betamethasone ointment | Methotrexate | |
| Conaghan *et al.* | Liarozole | Anti-tumor necrosis factor immunotherapy | Etretinate, psoralen ultraviolet A, hydroxyurea, methortrexate, cyclosporine A, prednisolone, sulfasalzaine, subcutaneous interferon alpha, intravenous gamma globulin, azathioprine, isotretinoin, acitretin |
| Behr *et al.* | Methotrexate and low dose acitretin | Methotrexate and low dose acitretin | Patient was initially on etretinate and responded well but has increased the dose without consultation and suffered toxicities |
| Nakafusa *et al.* | Vitamin $D_3$ ointment | Bucillamine and prednisolone | |
| Chan *et al.* | Methotrexate | Methotrexate | |

speculated by the authors that it may be the same mechanism that occurs in psoriatic arthritis given the close resemblance of PRP to psoriasis. Still, the pathophysiological relationship between psoriasis and seronegative arthritis is not well established.

It is well reported in the literature of the connection between myositis and PRP, specifically dermatomyositis. Dermatomyositis is an inflammatory myopathy of the proximal muscles with skin involvement. The skin involvement includes the classic findings of heliotrope rash, a purplish rash over the eyelid, Gottron papules, a scaly rash found over the knuckles of the hands, shawl sign, erythema over the shoulder areas exposed to the sun representing sun sensitivity. Dermatomyositis can be associated with internal malignancies.

Dermatomyositis with PRP is known as type Wong variant dermatomyositis, named after Wong who described a large series of patients from Hong Kong in 1969 (Requena *et al.*, 1997; Lupton *et al.*, 2000). However, it was believed that that PRP as a manifestation of dermatomyositis may have been reported as early as 1953 by O'Leary who did not describe or illustrate the histopathological findings (Requena *et al.*, 1997; Lupton *et al.*, 2000).

### TREATMENT

There are many different regimens to treat PRP. Fox *et al.* found over twenty different agents that have been used to treat PRP (Fox *et al.*, 1985). Many of these treatments had only variable or isolated success. As a result, no single agent is found to be reliably effective. It is difficult to treat a disorder when the etiology is unknown. Historically, there has been favorable response to vitamin A, systemically or topically. Unfortunately, the large oral doses required for effective treatment usually cause toxic side effects. The use of stanozolol is an attempt to increase circulating levels of retinol binding protein in order to increase tissue delivery of retinol (Cohen and Prystowsky, 1989). The androgenic side effects make it less desirable for women to use. To avoid the toxicity of vitamin A, synthetic vitamin A therapy has become popular with favorable clinical course and prognosis. Members of this class include isotretinoin and etretinate. It is estimated that 60–95% of patients will have clinical improvement within 6 months of starting therapy (Cohen and Prystowsky, 1989). Methotrexate has traditionally been a good second line treatment (Caplan *et al.*, 1997). Other cytotoxic and immunosuppressive medications, such as azathioprine and cyclosporine A, has been cited in the literature as successful modalities (Caplan *et al.*, 1997). Steroids appear to be effective but unpractical for long term use. Less conventional regimens include vitamin C and E, often used in combination with vitamin A, isoniazid, paraaminosalicyclic acid, penicillin, hydroxychloroquine, arsenic, pilocarpine, pituitary extract, foreign protein injections, and topical 6-aminonicotinamide (Cohen and Prystowsky, 1989).

### CONCLUSION

In summary, PRP is an extremely rare skin disorder. It is a member of the hyperkeratotic, papulosquamous diseases. There have been case reports citing rheumatologic associations, mainly arthritis and dermatomyositis. However, it is difficult to establish the precise rheumatologic association of PRP given the rarity of this disease. To really establish a relationship, there needs to be a large prospective study.

### *References*

Aguilar, A.R., Gomez, F., Balsa, F.T., Framil, J.P. and Oubina, P.N. (1973) "Pityriasis rubra pilaris with muscle and joint involvement", *Dermatologica* **146**, 361–366.

Albert, M.R. and Mackool, B.T. (1999) "Pityriasis rubra pilaris", *International Journal of Dermatology* **38**, 1–11.

Behr, F.D., Bangert, J.L. and Hansen, R.C. (2002) "Atypical pityriasis rubra pilaris associated arthropathy and osteoporosis: a case report with 15 year follow-up", *Pediatric Dermatology* **19**, 46–51.

Betloch, I., Ramon, R., Silvestre, J., Carnero, L., Albares, M. and Banuls, J. (2001) "Acute juvenile pityriasis rubra pilaris: a superantigen mediated disease?", *Pediatric Dermatology* **18**, 411–414.

Bonomo, R.A., Korman, N., Nagashima-Whalen, L., Briggs, J., Graham, R. and Salata, R. (1997) "Pityriasis rubra pilaris: an unusual cutaneous complication of AIDS", *American Journal of Medical Sciences* **314**, 118–121.

Caplan, S.E., Lowitt, M.H. and Kao, G.F. (1997) "Early presentation of pityriasis rubra pilaris", *Cutis* **60**, 291–296.

Cohen, P.R. and Prystowsky, J. (1989) "Pityriasis rubra pilaris: a review of diagnosis and treatment", *Journal of the American Academy of Dermatology* **20**, 801–807.

Conaghan, P.G., Sommer, S., McGonagle, D., Veale, D., Waldmann, H., Hale, G., *et al.* (1999) "The relationship between pityriasis rubra pilaris and inflammatory arthritis", *Arthritis & Rheumatism* **42**, 1998–2001.

Duke, R.A., Barrett, M.R., Salazar, J.E., Scott, R.L. and Sebes, J.E. (1987) "Acro-osteolysis secondary to pityriasis rubra pilaris", *American Journal of Roentgenology* **149**, 1082–1083.

Fiallo, P., Tagliapietra, A.G. and Santoro, G. (1996) "Arthropathic pityriasis rubra pilaris", *British Journal of Dermatology* **134**, 1154–1155.

Fox, B., Captain, M.C., Odom, R.B., *et al.* (1985) "Papulosquamous diseases: a review", *Journal of the American Academy of Dermatology* **12**, 597–624.

Griffiths, W.D. (1976) "Pityriasis rubra pilaris—an historical approach", *Clinical and Experimental Dermatology* **1**, 37–50.

Griffiths, W.D. (1980) "Pityriasis rubra pilaris", *Clinical and Experimental Dermatology* **5**, 105–112.

Lister, R.K., Perry, J.D. and Cerio, R. (1997) "Pityriasis rubra pilaris and a seronegative polyarthritis", *British Journal of Dermatology* **137**, 318–319.

Lupton, J.R., Figueroa, P., Berberian, B.J. and Sulica, V.I. (2000) "An unusual presentation of dermatomyositis: the type Wong variant revisited", *Journal of the American Academy of Dermatology* **43**, 908–912.

Miralles, E.S., Nunez, M., De Las Heras, M.E., Perez, B., Moreno, R. and Ledo, A. (1995) "Pityriasis rubra pilaris and human immunodeficiency virus infection", *British Journal of Dermatology* **133**, 990–993.

Misery, L., Faure, M. and Claudy, A. (1996) "Pityriasis rubra pilaris and human immunodeficiency virus infection-type 6 pityriasis rubra pilaris?", *British Journal of Dermatology* **135**, 1008–1009.

Nakafusa, J., Misago, N. and Narisawa, Y. (2002) "Pityriasis rubra pilaris in association with polyarthritis", *Dermatology* **205**, 298–300.

Requena, L., Grilli, R., Soriano, L., Escalonilla, P., Farina, C. and Martin, L. (1997) "Dermatomyositis with a pityriasis rubra pilaris-like eruption: a little-known distinctive cutaneous manifestation of dermatomyositis", *British Journal of Dermatology* **136**, 768–771.

Rinker, M.H. and Shenefelt, P.D. (2002, May 8). Pityriasis rubra pilaris. *eMedicine*. Retrieved January 6, (2003), from http://www.emedicine.com/DERM/topic337.htm.

